# Tic disorder in allergic rhinitis children and adolescents: a case-control study

**DOI:** 10.1186/s12887-023-04482-4

**Published:** 2024-01-05

**Authors:** Hossein Esmaeilzadeh, Mohammad Reza Yousefi, Negar Mortazavi, Mohammad Amin Gholami, Mohebat Vali, Seyed Ali Dastgheib

**Affiliations:** 1 Allergy Research Center, Shiraz University of Medical Sciences, Shiraz, Iran; 2 Department of Allergy and Clinical Immunology, Namazi Hospital, Shiraz University of Medical Sciences, Shiraz, Iran; 3 MD/MPH Program, School of Medicine, Shiraz University of Medical Sciences, Shiraz, Iran; 4 Department of Clinical Pharmacy, School of Pharmacy, Shiraz University of Medical Sciences, Shiraz, Iran; 5 Student Research Committee, Shiraz University of Medical Sciences, Shiraz, Iran; 6 Research Center for Psychiatry and Behavioral Sciences, Shiraz University of Medical Sciences, Shiraz, Iran

**Keywords:** Rhinitis, Allergic, Tics, Motor tic, Vocal tic, Child, Adolescent

## Abstract

**Background:**

Allergic rhinitis is the most common allergic disease. It can accompany psychological disorders such as tic disorders due to the prolonged course of the symptoms of allergic rhinitis. This pioneer case-control study aims to investigate tic disorders in children and adolescents under 18 years of age diagnosed with allergic rhinitis.

**Method:**

The case group in this study consisted of patients who had both allergic rhinitis and tic disorders. Patients with allergic rhinitis without tic disorders were also enrolled as the control group with matched gender and age. Demographic characteristics, tic classifications, and contributing factors for allergic rhinitis and tic disorders were studied among the cases. Tic disorders were evaluated using DSM-5 criteria for the classification of tic disorders.

**Results:**

47 patients in the case group and 47 patients in the control group were included in this study. 53.2% and 46.8% were males and females in the case group, respectively. The mean age of the patients was 10.46 ± 3.97 years old. Sound tics were more common among the patients compared to motor tics. Patients with concomitant AR and tic disorders had more days per week with AR symptoms (P-value ≤ 0.001; OR (every day vs. three days a week = 11.02(2.98, 40.76))). Most patients with sound tick were women (p: 0.026), and most patients with motion tic were in the Provisional tic disorder group (p: 0.001). The history of infantile eczema was seen more in patients without sound tic (p: 0.025), and otitis media was significantly less common among patients with sound tics (p: 0.026). Provisional tic disorder was the most common class among the patients. In the case group (coexistence between allergic rhinitis and tic) compared to the control group, patients had significantly more days with AR symptoms per week.

**Conclusion:**

This preliminary study indicates that Provisional tic disorder was the most common classification of tic among patients with allergic rhinitis, especially in patients with motor tics. Asthma in motor tics, a history of food allergy in infancy, and a history of infantile eczema were also common among patients with vocal tics. Also, patients with allergic rhinitis and tic had more severe disease (more symptoms per week) than those with rhinitis alone. These findings emphasize the association of tic disorders with immunological pathways.

## Background

Allergic rhinitis is one of the most common allergic diseases and one of the most common non-communicable diseases worldwide, which can coincide with other diseases, such as tic disorders [1, 2].

Tic disorders are defined as involuntary or semi-voluntary motor movements (motor tics) or sound production (vocal tics or Tourette syndrome). Tics are usually sudden, brief, repetitive, rapid, meaningless, and unpredictable symptoms that typically develop in childhood [3, 4]. 5th edition of the Diagnostic and Statistical Manual of Mental Disorders (DSM-5) published by the American Psychiatric Association classifies Neurologic tic into three distinct conditions: Tourette syndrome, Persistent (Chronic) Motor or Vocal Tic Disorder, and provisional (transient) tic disorder [4, 5]. Several studies have reported a connection between allergic conditions and tic disorders [2, 6, 7]. The etiology of tic disorders is complex and may be due to the interaction of genetic, environmental, immunological, and hormonal factors [8]. In patients with tic disorder, disorders in gene expression profile related to immune function, changes in immune cell subsets, immune phenotypes, and effectors have been previously reported [9]. Environmental triggers that activate the immune system, such as pathogenic infections and allergens, have also been suggested to play a role in childhood tic disorder [10].

Anxiety and mood disorders are believed to be the predisposing factors for tic disorders [11]. Regarding AR, factors such as a history of Asthma, tobacco exposure, and aeroallergens exposure have been shown as risk factors [12]. Also, risk factors such as male sex, firstborn status, and early use of antibiotics are described for allergic rhinitis [13]. The severity of allergic rhinitis is also considered a factor that could play a role in the association of AR and tic disorders [14]. Previous research has suggested [2, 6, 7] that allergic rhinitis can affect people simultaneously with tic disorder in children and adolescents. The chronicity of patients’ symptoms leads to changes in their behavior and quality of life, which in turn causes neurological and psychological disorders in the long run. This study is an effort to investigate the relationship between allergic rhinitis and tic disorders in children and adolescents who have experienced repetitive behaviors even in the absence of allergens. The authors hypothesize that certain chronicity and severity of allergic rhinitis symptoms, accompanied by several contributing factors, can eventually lead to tic-like behaviors. The authors aim to investigate which classification of tic disorders is more prominent among patients with concomitant allergic rhinitis and tic disorders and what contributing factors are responsible for this association. Determining this issue can lead to a better understanding of the relationship between these conditions and the cause of each of them. Therefore, this interdisciplinary study between psychiatry and clinical immunology aims to investigate the association between tic disorders and allergic rhinitis in children and adolescents and identify the underlying contributing factors.

## Method

### Study design

This study is designed as a case-control study to evaluate the association and contributing factors of allergic rhinitis and neurological tic disorders in children and adolescents under 18 years old, referring to a tertiary referral center in southwest Iran in 2021–2022. The data used in this study is collected by interviewing the patients or their legal guardians referred to the asthma-allergy and clinical immunology clinic in south Iran due to allergic rhinitis symptoms. The sampling method is non-randomized convenience sampling. All the data is confidential.

### Data gathering

A specialized pediatrician in allergy and clinical immunology evaluated all the cases in this study. The diagnosis of allergic rhinitis was based on ARIA guidelines [15] and a comprehensive examination of the patient’s medical history and physical symptoms, as well as skin testing (wheal more than 3 mm or flare more than 10 mm in comparison to negative control is considered as a positive result). Patients diagnosed with allergic rhinitis who exhibited signs or symptoms of tic disorders (motor tics such as blinking or shoulder shrugging or vocal tics such as humming, sniffing, clearing the throat, or yelling out a word or phrase) were referred to a psychiatrist for further evaluation and diagnosis. The diagnosis and classification of tic disorders were based on the Diagnostic and Statistical Manual of Mental Disorders, 5th Edition (DSM-5) [4]. Additionally, patients diagnosed with allergic rhinitis but did not exhibit any symptoms of tic disorders were included in the study as a control group, with age and sex frequency matching. The collected data include demographic characteristics such as age, sex, date of birth, place of residence, and place of birth, as well as family history of other diseases, whether the patient is a first-born, age of onset and duration of AR and tic disorders, smoking habits of the mother during and after pregnancy, frequency of AR and tic disorder symptoms, detailed history of other diseases (e.g., migraine, cold, etc.), detailed history of other allergic conditions (e.g., food allergy, eczema, etc.), detailed history of neurological, psychological, or behavioral disorders, and detailed drug history. The classification of tic disorders was based on the DSM-5 criteria, and the patient’s laboratory workup, including skin prick testing, was collected and evaluated. After considering our assumption and previous studies, we have identified the following variables as predictors:

- Age of onset and duration of AR and tic disorders.
- Smoking habits of mother during and after pregnancy.
- Family history of other diseases.
- Detailed history of other allergic conditions.
- Detailed history of neurological, psychological, or behavioral disorders.
- Detailed drug history.

Also, the variables Age, Sex, living place, Birthplace, Family history of other diseases, Detailed history of other diseases, Detailed history of other allergic conditions, Detailed history of neurological, psychological, or behavioral disorders, and Detailed drug history were considered as potential confounders. The frequency of AR and tic disorder symptoms and Skin prick testing variables were considered effect modifiers.

### Inclusion and exclusion criteria

This study enrolled all the patients who fulfilled the following criteria in the case group: children and adolescents under 18 years old, diagnosed with allergic rhinitis, exhibited symptoms of tic disorders, and were referred to a tertiary referral center in southwest Iran from 2021 to 2022. Furthermore, all the patients with the following criteria were enrolled in the control group: children and adolescents under 18 years old, diagnosed with allergic rhinitis, without any symptoms of tic disorders or repetitive behaviors, and were referred to a tertiary referral center in the southwest of Iran from 2021 to 2022. The exclusion criteria included patients above the age of 18, patients who passed away during the study, patients who decided to leave the study, patients whose diagnosis was confirmed as something other than allergic rhinitis, and patients with conditions such as Huntington’s disease, epilepsy, and post-viral encephalitis. Patients taking medication that could cause or exhibit tic-like symptoms during sleep were also excluded.

### S**tatistical analysis**

The sample size of this study was determined based on recent research [7], using the statistics and sample size application to “compare two proportions” Formula. The following settings were used: proportion in group 1 = 0.406, proportion in group 2 = 0.15, alpha error probability of 5%, power of 80%, and a ratio of 1 between the case and control groups. The final total sample size was 47 patients in each group. The normal distribution of the data was assessed using the Kolmogorov-Smirnov test. The mean and standard deviation were reported for quantitative data, and the number and percentage were reported for qualitative data. Independent t-tests (parametric tests), Mann-Whitney tests (non-parametric tests), and chi-square tests were used for each quantitative variable. Also, logistic regression was performed to compare the target and control groups. In univariate logistic regression, variables with a p-value less than 0.2 remained in the model, and then multivariable logistic regression was run using the forward method. A p-value of less than 0.05 was considered significant. The data were analyzed using version 22.0 of the SPSS program.

## Results

In total, 47 patients were enrolled in each group. In the case group, 53.2% of the cases were male, and 46.8% were female. The mean age of the participants in the case group was 10.46 ± 3.97 years old. There was no statistically significant difference in age between the group with motor tics and the group without motor tics (P-value = 0.732). Similarly, there was no significant difference between the two groups with and without vocal tics (P-value = 0.368).

The age of onset for allergic rhinitis did not show a statistically significant difference between the two groups with and without motor tics (P-value = 0.864). Additionally, there was no statistically significant difference in the age of onset for allergic rhinitis between the two groups with vocal tics and without vocal tics (P-value = 0.350). (Table 1) Most people with motor tics are men (11 people or 68.75%), but there was no statistical difference in gender between people with and without motor tics (P-value = 0.125). However, most of the patients with vocal tics are women (22 or 52.38%), and there was a significant statistical difference between the two groups with and without vocal tics regarding gender (P-value = 0.026). (See Table 1) Regarding the classification of tic disorders, Most patients with motor tics were in the Provisional tic disorder group (P-value = 0.001).

The history of infantile eczema differed between the two groups with and without vocal tics, with infantile eczema being more prevalent in patients without vocal tics (n = 35 or 83.33%). Additionally, food allergy in infancy was statistically significant between the two groups with and without motor tics (P-value = 0.022) and with and without vocal tics (P-value = 0.024). The history of middle ear infection was significantly less common among patients with sound tics (P-value = 0.006) (See Table 1).

**Fig. 1:**
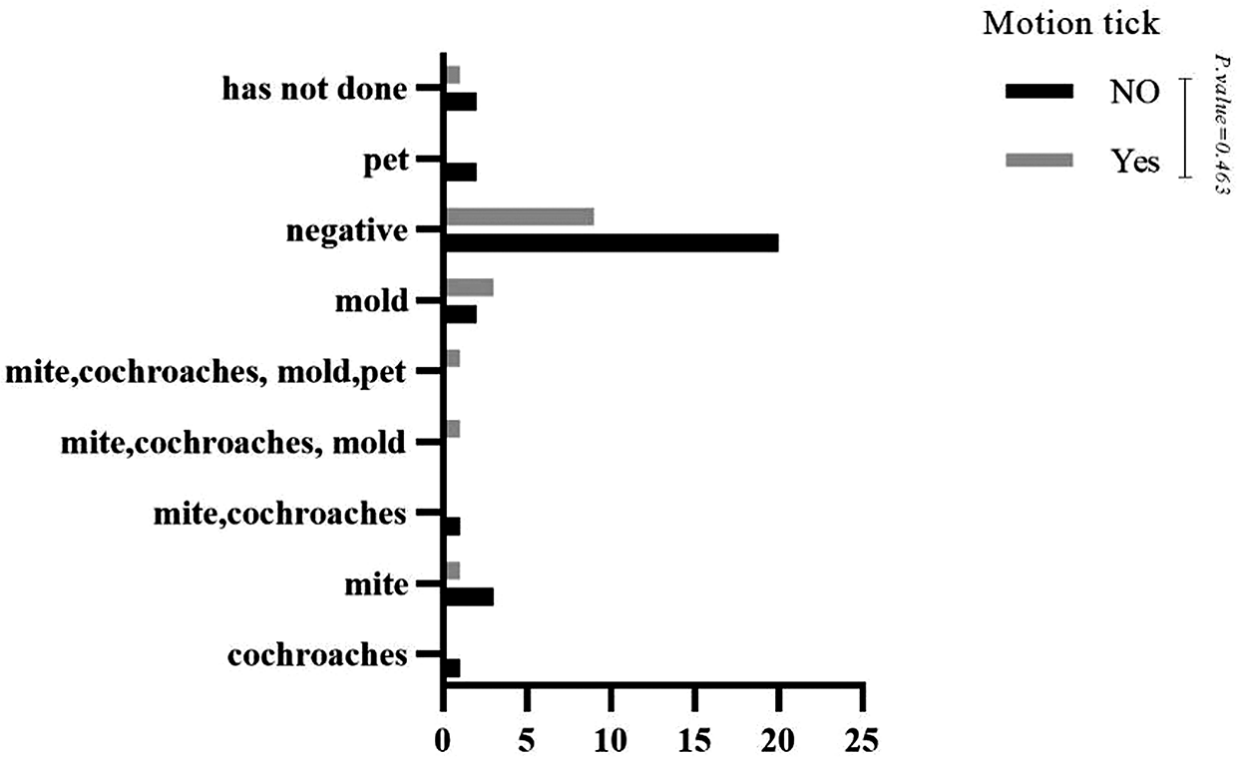
Comparison of skin prick test results with indoor aeroallergen in patients with and without motion tics

**Fig. 2:**
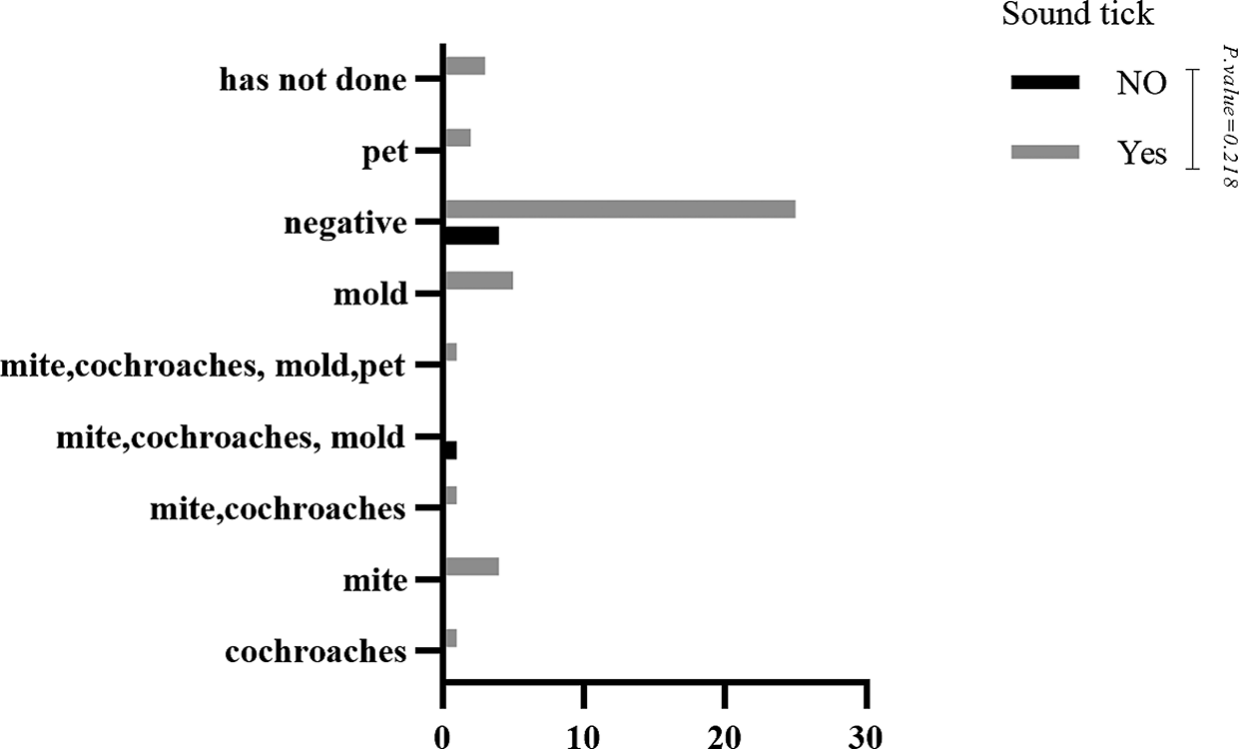
Comparison of skin prick test results with indoor aeroallergen in patients with and without sound tics

**Fig. 3:**
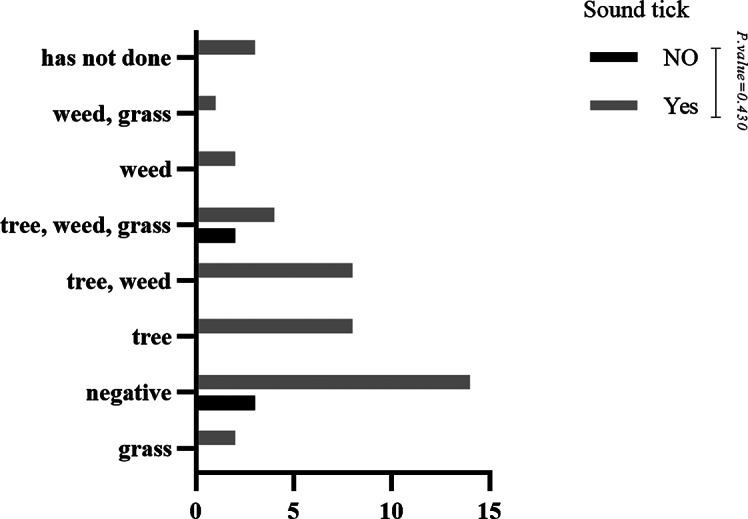
Comparison of skin prick test results with outdoor aeroallergen in patients with and without sound tics

**Fig. 4:**
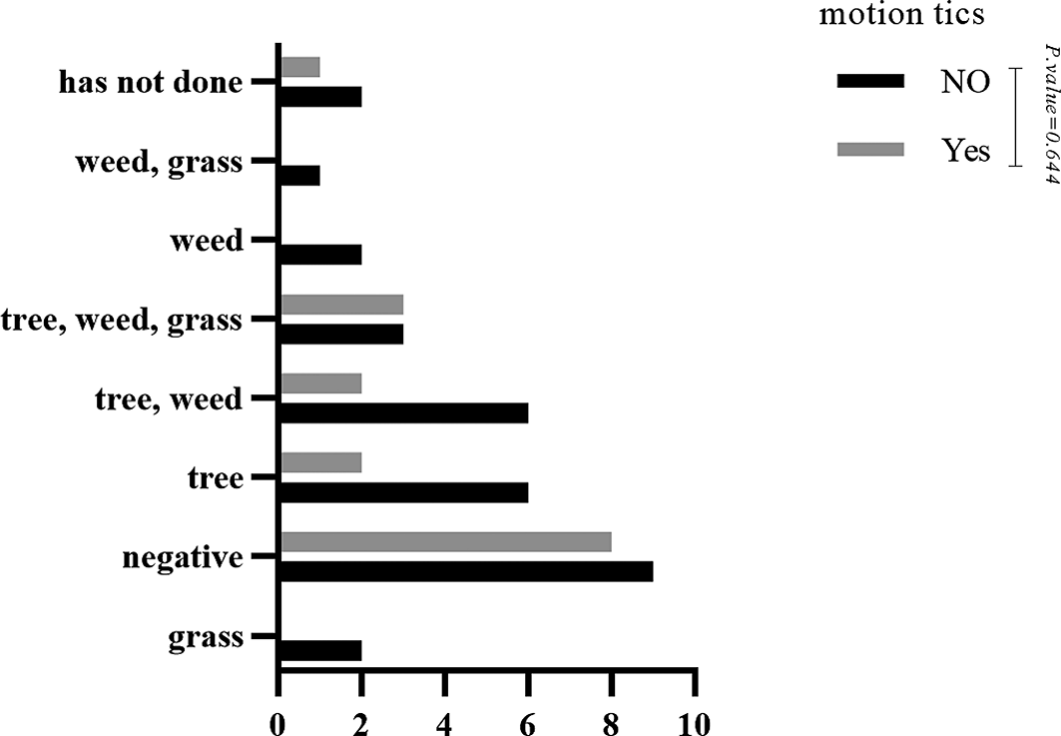
Comparison of skin prick test results with outdoor aeroallergen in patients with and without motion tics

No statistically significant difference was observed when comparing the skin prick test results with indoor aeroallergens in patients with and without motor tics (P-value = 0.463) (See Fig. 1). However, most skin prick test results with indoor aeroallergens were negative, followed by mold. In the case of vocal tics, the comparison of skin prick test results with indoor aeroallergens was similar to motor tics (P-value = 0.218) (See Fig. 2). No statistically significant difference was observed when comparing the skin prick test results with outdoor aeroallergens in patients with and without vocal tics (P-value = 0.430) (See Fig. 3). Most skin prick test results with outdoor aeroallergens were negative, followed by trees and weeds. Additionally, regarding skin prick test results with outdoor aeroallergens, no statistically significant difference was observed in motor tics (P-value = 0.644) (See Fig. 4), and most of the tests were negative, followed by trees, weeds, and grass.

Table 2 shows the comparison between the case and control groups. Asthma was overall more common among the control group (P-value = 0.012; OR = 0.34(0.14,0.80)). Episodes of the common cold (P-value = 0.012; OR = 3.30(1.26,8.62)) and night cough during those episodes (P-value = 0.059; OR = 2.22(0.96,5.14)) were significantly more frequent in the case group. Patients in the case group experienced AR symptoms more days per week (P-value ≤ 0.001; OR (every day vs. three days a week) = 11.02(2.98, 40.76)) and took more medication for AR (P-value = 0.0113; OR = 5.02(1.31,19.21)). The variable age of asthma onset (P-value ≤ 0.001) also showed statistically significant differences between the case and control groups.

**Table 1:** Demographic, clinical, and laboratory findings (motion tic vs. sound tic)

Variable		Motion tick		P.value^*^	Sound tick		P.value^*^
		Yes	No		Yes	No	
Age, Mean ± SD		10.18 ± 3.48	10.61 ± 4.25	0.732	10.28 ± 3.38	12.00 ± 4.89	0.368
Age of allergic rhinitis, Mean ± SD		3.86 ± 0.96	3.98 ± 0.71	0.864	7.45 ± 3.76	9.20 ± 5.16	0.350
The duration of allergic rhinitis (month), Median(IQR)		24(15,45)	24(6,48)	0.396	24(7.5,48)	36(18,48)	0.465
Age of onset of infantile eczema (month), Median(IQR)		3(1.5, 24)	2(1,.)	0.250	2(1.5, 7.5)	2(1,.)	0.999
Duration of infantile eczema (month), Median(IQR)		3(1.12, 14)	6(3,.)	0.393	4(1.62, 9)	3(2,.)	0.999
Age of asthma onset (year), Mean ± SD		3.41 ± 1.20	3.91 ± 1.74	0.943	7.00 ± 2.89	11.50 ± 4.94	0.089
Age of resolution of infancy allergy (year), Median(IQR)		6.5(6,9.5)	10(4,10)	0.550	3(1,.)	Not report	Not report
The age of sinusitis resolution (year), Mean ± SD		5.00±.	4.5 ± 0.70	0.667	4.50 ± 0.70	5.00±.	0.667
Age of tick onset (year), Mean ± SD		8.75 ± 3.69	9.79 ± 4.24	0.411	9.20 ± 4.03	11.40 ± 4.15	0.257
Sex, N (%)	Female	5(22.7)	17(77.3)	0.125	22(100.0)	0(0.0)	0.026
	Male	11(44.0)	14(56.0)		20(80.0)	5(20.0)	
Classification of tic disorders, N (%)	Persistent motor tic disorder	1(50.0)	1(50.0)	0.001	1(50.0)	1(50.0)	0.239
	Persistent vocal tic disorder	0(0.0)	1(100.0)		1(100.0)	0(0.0)	
	Provisional tic disorder	8(21.6)	29(78.4)		33(89.2)	4(10.8)	
	Tourette syndrome	7(100.0)	0(0.0)		7(100.0)	0(0.0)	
Location, N(%)	Village	3(37.5)	5(62.5)	0.627	6(75.0)	2(25.0)	0.178
	big city	7(28.0)	18(72.0)		22(88.0)	3(12.0)	
	small city	6(42.9)	8(57.1)		14(100.0)	0(0.0)	
The first child of the family, N (%)		8(28.6)	20(71.4)	0.337	25(89.3)	3(10.7)	0.984
Family history of allergies, N (%)		9(37.5)	15(62.5)	0.609	22(91.0)	2(8.3)	0.601
Antibiotic use in early life, N (%)		9(33.3)	18(66.7)	0.905	25(92.6)	2(7.4)	0.404
The history of passive exposure of the mother to cigarette smoke during pregnancy, N (%)		1(16.7)	5(83.3)	0.336	6(100.0)	0(0.0)	0.366
The history of passive exposure of the mother to cigarette smoke in the first year of life, N (%)		1(16.7)	5(83.3)	0.336	6(100.0)	0(0.0)	0.366
Mother’s smoking during pregnancy, N (%)		0(0.0)	1(100.0)	0.468	1(100.0)	0(0.0)	0.727
Preterm birth, N (%)		2(40.0)	3(60.0)	0.766	4(80.0)	1(20.0)	0.473
Day of the week AR symptoms, N (%)	3 days a week	2(66.7)	1(33.3)	0.162	2(66.7)	1(33.3)	0.401
	4 days a week	1(100.0)	0(0.0)		1(100.0)	0(0.0)	
	Every day	13(30.2)	30(69.8)		39(90.7)	4(9.3)	
Sleep disturbance due to AR, N (%)		8(44.4)	10(55.6)	0.236	15(83.3)	3(16.7)	0.291
Disruption at work or school due to AR, N (%)		2(15.4)	11(84.6)	0.095	13(100.0)	0(0.0)	0.144
History of other allergic diseases, N (%)		3(33.3)	6(66.7)	0.960	8(88.9)	1(11.1)	0.959
History of infantile eczema, N (%)		5(50.0)	5(50.0)	0.230	7(70.0)	3(30.0)	0.025
Associated with asthma, N (%)		8(53.3)	7(46.7)	0.056	13(86.7)	2(13.3)	0.682
Seasonal exacerbation of AR, N (%)		6(25.0)	18(75.0)	0.181	22(91.7)	2(8.3)	0.601
Frequent colds, N (%)		8(42.1)	11(57.9)	0.337	16(84.2)	3(15.8)	0.342
Night cough in colds, N (%)		8(33.3)	16(66.7)	0.917	21(87.5)	3(12.5)	0.672
Active cough in colds, N (%)		10(35.7)	18(64.3)	0.769	25(89.3)	3(10.7)	0.984
Active cough without a cold, N (%)		8(44.4)	10(55.6)	0.236	16(88.9)	2(11.1)	0.934
Food allergy in infancy, N (%)		4(80.0)	1(20.0)	0.022	3(60.0)	2(40.0)	0.024
Taking medication for AR, N (%)		16(36.4)	28(63.6)	0.198	39(88.6)	5(11.4)	0.535
Improvement of AR symptoms with medication, N (%)		15(36.6)	26(63.4)	0.910	37(90.2)	4(9.8)	0.214
Improvement of tic symptoms with improvement of AR symptoms, N (%)		12(34.3)	23(65.7)	0.572	32(91.4)	3(8.6)	0.250
Worsening of tic symptoms with worsening of AR symptoms, N (%)		15(37.5)	25(62.5)	0.232	35(87.5)	5(12.5)	0.322
History of sinusitis, N (%)		3(50.0)	3(50.0)	0.377	4(66.7)	2(33.3)	0.054
History of conjunctivitis, N (%)		4(57.1)	3(42.9)	0.162	5(71.4)	2(28.6)	0.095
History of middle ear infection, N (%)		6(46.2)	7(53.8)	0.279	9(69.2)	4(30.8)	0.006
The presence of ticks before the onset of AR symptoms, N (%)		4(66.7)	2(33.3)	0.071	6(100.0)	0(0.0)	0.366
History of other mental and neurological diseases, N (%)		1(50.0)	1(50.0)	0.626	1(50.0)	1(50.0)	0.065
History of systemic diseases, N (%)		1(20.0)	4(80.0)	0.483	5(100.0)	0(0.0)	0.414
Drug history, N (%)		0(0.0)	3(100.0)	0.198	3(100.0)	0(0.0)	0.537
Treatment for tics, N (%)		6(85.7)	1(14.3)	0.002	7(100.0)	0(0.0)	0.322
Taking the drug at the same time as the tic, N (%)		5(83.3)	1(16.7)	0.065	3(100.0)	0(0.0)	0.537

SD: Standard Deviation

IQR: interquartile range

AR: rheumatoid arthritis

^*^ Comparison of Tic Disorders, Use of Independent t test(parametric) or Mann–Whitney U test(non-parametric) OR Chi square test

**Table 2:** Demographic, clinical, and laboratory findings for comparison of case and control group

Variable		Case Group (Accompanying allergic rhinitis and tic) N = 47	Control Group (Allergic rhinitis alone) N = 47	P.value^*^
Age, Mean ± SD		10.46 ± 3.97	9.27 ± 3.18	0.112
Age of allergic rhinitis, Mean ± SD		7.63 ± 3.90	6.41 ± 4.12	0.143
Age of asthma onset (year), Mean ± SD		7.69 ± 3.44	4.75 ± 2.86	≤ 0.001
Sex, N (%)	Female	22(46.8)	19(40.42)	0.532
	Male	25(53.2)	28(59.58)	
The first child of the family, N (%)		28(59.6)	30(63.83)	0.671
Family history of allergies, N (%)		24(51.1)	26(55.33)	0.679
Antibiotic use in early life, N (%)		27(57.4)	25(53.20)	0.678
The history of passive exposure of the mother to cigarette smoke in the first year of life, N (%)		6(12.8)	8(17.03)	0.562
Mother’s smoking during pregnancy, N (%)		1(2.1)	2(4.26)	0.557
Preterm birth, N (%)		5(10.6)	8(17.03)	0.370
Day of the week AR symptoms, N (%)	3 days a week	3(6.4)	20(42.55)	≤ 0.001
	4 days a week	1(2.1)	1(2.1)	
	Every day	43(91.5)	26(55.35)	
Sleep disturbance due to AR, N (%)		18(38.3)	17(36.18)	0.831
Disruption at work or school due to AR, N (%)		13(27.7)	8(17.03)	0.215
History of infantile eczema, N (%)		10(21.3)	8(17.03)	0.600
Associated with asthma, N (%)		15(31.9)	27(57.45)	0.012
Seasonal exacerbation of AR, N (%)		24(51.1)	32(68.09)	0.092
Frequent colds, N (%)		19(40.4)	8(17.03)	0.012
Night cough in colds, N (%)		24(51.1)	15(31.92)	0.059
Active cough in colds, N (%)		28(59.6)	27(57.45)	0.834
Active cough without a cold, N (%)		18(38.3)	13(27.66)	0.272
Food allergy in infancy, N (%)		5(10.6)	9(19.15)	0.246
Taking medication for AR, N (%)		44(93.6)	35(74.47)	0.0113
Improvement of AR symptoms with medication, N (%)		41(93.2)	45(95.74)	0.591
History of sinusitis, N (%)		6(12.8)	12(25.54)	0.115
History of conjunctivitis, N (%)		7(14.9)	8(17.03)	0.778
History of middle ear infection, N (%)		13(27.7)	20(42.56)	0.130
History of other mental and neurological diseases, N (%)		2(4.3)	1(2.13)	0.557
Drug history, N (%)		3(6.4)	2(4.26)	0.645

SD: Standard Deviation

IQR: interquartile range

AR: rheumatoid arthritis

^*^ Comparison of case and control group, Use of Independent t test(parametric) or Mann–Whitney U test(non-parametric) OR Chi square test

## Discussion

Given the high prevalence of allergic rhinitis among children and adolescents, conducting a thorough study of its comorbidities, such as tic disorders, is essential. This literature is pioneering in discussing multiple contributing factors associated with the concomitance of these diseases to provide a deeper understanding. Previous studies have shown that allergic conditions, including allergic rhinitis, are associated with tic disorders [2, 6, 7]. Gender, classification of tic disorder, history of infantile eczema, history of food allergy in infancy, and otitis media have been proven to have a significant difference among patients with motor versus vocal tics with comorbid allergic rhinitis. Age of asthma onset, days per week with AR symptoms, association with asthma, frequent colds, night cough in colds, and taking medication for AR showed statistically significant differences between the case and control groups. Among the demographic characteristics of the study population, only gender has shown a significant difference between patients with motor and vocal tics; vocal tics are more common among females, with all of them presenting with at least one type of vocal tic. No specific reason could be found for this finding, and there are no similar demographic studies to the best of the author’s knowledge. Patients with motor tics were mostly males (11 males versus five female cases), and no statistical significance was observed.

This study indicates that the most prevalent tic disorder among patients with allergic rhinitis is provisional tic disorder, with 78.7% of all cases diagnosed with this type of tic disorder. This finding supports the work of Jiehong Huang et al. (2021), who demonstrated that allergic diseases are associated with tic disorders, especially provisional tic disorder [6]. There is a significant difference between patients with sound and motion tics; most patients with at least one motion tic are diagnosed with provisional tic disorder. However, sound tics are more common among patients than motor tics (80.8% versus 34.04%, respectively).

Regarding the neurological basis of allergic rhinitis, it has been shown that the nervous system plays an essential role in regulating the main symptoms of rhinitis, such as congestion, mucus secretion, and sneezing [16, 17]. The activation of mast cells and other immune cells results in the secretion of immune mediators such as interleukins, tumor necrosis factor-alpha, and interferon-gamma. These immune mediators, in turn, activate neural pathways that generate the common symptoms seen in allergic rhinitis [18]. When exposed to allergens, patients with seasonal allergic rhinitis tend to have an exaggerated neural response. A study even suggested that the nasal mucosa of children with allergic rhinitis is hyper-innervated, which could be the reason for neural hyperactivity in allergic rhinitis [19].

The median duration of allergic rhinitis in the patients was 24 months, indicating the chronic nature of the disease, and all patients experienced moderate to severe symptoms of allergic rhinitis. These findings further support the idea that chronic behaviors, such as sniffing, coughing, and clearing the throat, could, over time, develop into tic disorders and profoundly affect patients’ quality of life. Further case-control studies are necessary to confirm this hypothesis. Only 21.3% of the study population had a history of infantile eczema. The results suggest that patients with sound tics had a significantly lower infantile eczema rate than the other group. Food allergy in infancy was a relatively infrequent presentation among the study groups. A significant finding was that most patients with sound tics had a lower prevalence of infantile food allergy. Roughly 32% of the cases had positive spirometry tests and clinical manifestations of asthma, although no significant difference was observed between patients with sound and motor tics. These results show a higher prevalence of asthma among the study groups compared to previous studies, which indicated that 18.2% of their cases had comorbid tic disorders and asthma [18]. These findings further support the previous results regarding an association between tic disorders and immunological abnormalities. Ying Li et al. conducted a meta-analysis in 2022 to indicate changes in proinflammatory cytokine patterns and T cells in pediatric patients with Tourette syndrome. They proved an association between peripheral immune system activation and Tourette syndrome [20]. Another study conducted in 2010 has shown that interleukin-2 (IL-2) concentrations are associated with the severity of tic disorders [21]. In that regard, IL-2 has significantly increased allergic airway responses [22].

A history of sinusitis, conjunctivitis, and middle ear infection was present in 12.8%, 14.9%, and 27.7% of the cases, respectively. The history of middle ear infection was significantly less common among patients with sound tics. The prevalence of these conditions among patients with tic disorders is lower than in previous studies [18]. These differences could result from genetic or racial variations among the study populations. 57.4% of all cases had a history of infection in the early stages of their lives, which required an antibiotic prescription. Previous studies have shown that early exposure to infections could increase the risk of tic disorders later in patients’ lives [23]. A nationwide cohort study of 14,024 children and adolescents conducted in Taiwan between 1997 and 2012 indicated that after bacterial infections, the risk for major mental disorders, including tic disorders, increases among the cases [24]. Further investigations are required to shed light on the exact mechanisms of this association and its immunological aspect.

Of all the patients in this study, 38.3% had positive skin prick tests for indoor aeroallergens, with mold being the most common indoor aeroallergen. Of all the patients, 63.8% had positive skin prick tests for outdoor aeroallergens, with tree and weed being more common among the positive skin prick test results. No significant difference was found between patients with and without sound or motion tics. A previous study has shown that nearly 22% of their cases who had Tourette syndrome presented with positive skin prick tests [17]. Regarding the immunological basis of tic disorders, animal studies have shown evidence of immune dysfunction in Tourette syndrome, but it is still poorly understood [25]. These findings are also coherent with immunologic pathways involved in patients with tic disorders and require more detailed studies. All the patients included in this study had been diagnosed with moderate to severe allergic rhinitis and prescribed medication for that. 93.6% of the cases had used the medication, and 93.2% had reported significant improvement in their symptoms and daily life quality. No significant difference was observed between the two groups in this sense. Nearly 85% of the patients reported worsening of their tic symptoms and their allergic rhinitis symptoms.

Moreover, 79.5% of the cases reported improvement of their tic symptoms with an improvement of their allergic symptoms, in a sense that only 14.9% of the cases (7 cases) required medical treatment for their tic disorder, of whom six patients had been diagnosed with Tourette syndrome and had been prescribed medication prior to allergic rhinitis diagnosis. These findings indicate a close association between these two conditions. Furthermore, only 12.8% of the cases reported having tic presentations before being diagnosed with allergic rhinitis. Others had shown tic-like presentations only after being diagnosed with allergic rhinitis and, later on, have been diagnosed with tic disorder (mostly provisional tic disorder). These findings support the hypothesis, as mentioned earlier, that chronic and severe allergic symptoms could cause or aggravate tic symptoms; therefore, alleviating allergic symptoms in the patients could have improved their tic symptoms. Further comprehensive studies are required to prove this.

Compared to the control group, patients with concomitant AR and tic disorders presented mostly with persistent allergic rhinitis according to the ARIA classification of AR symptoms, with more than four days per week of the presence of the symptoms [15]. The Odds ratio of days with AR symptoms during a week was 55.42, which shows a great significance between the case and control groups. Patients with AR symptoms every day per week had more chance of developing concomitant tic disorders and Allergic rhinitis compared to patients with 3 or 4 symptomatic days per week. Patients in the case group also took significantly more medication to relieve their AR symptoms. These novel findings further prove the frequency of the symptoms in the case group. They favor the central hypothesis of this study that the frequency and severity of AR symptoms could lead to the development of involuntary repetitive movements and tic disorders.

Episodes of common cold and night cough during those episodes were significantly more frequent among patients with both tic and AR. Also, with OR = 6.02, it is significantly more possible for patients with frequent cold episodes to develop concomitant AR and tic disorders. This finding is parallel to those of Hoekstra et al., who showed in 2005 that exacerbation of tic symptoms in children is associated with the common cold [26]. This association can be the result of possible immune factors and pathways involved in the development of tic disorder and could be proven by immunological studies of these groups.

Asthma was more common among the control group, and patients with concomitant allergic rhinitis and tic disorder had higher age of onset of asthma symptoms. The odd ratio for association with asthma was 0.02, which indicates that patients with asthma had lower chances of developing concomitant tic disorders and AR compared to the control group. This could indicate an association between late-onset asthma and tic disorders. Previous studies [2] have shown that Asthma had an association with comorbid tic disorders and ADHD, which is slightly different from the findings of the current study. These differences could be due to methodologic (evaluating patients with the dual diagnosis of tic disorders and ADHD in a single group in previous studies [2]) and demographic variations. Further studies are required to shed light on this issue.

### Strengths and limitations

This study is pioneering, as few studies have investigated tic disorder as a comorbidity of allergic rhinitis and explored the risk factors of such co-occurrence. The limitations of this study are the scarcity of cases with concomitant allergic rhinitis and tic disorders, as well as the lack of similar studies. Further case-control studies with more participants are needed to explore more risk factors over prolonged periods.

## Conclusion

This preliminary study investigated the contributing factors and association of allergic rhinitis and tic disorders. The results suggest that provisional tic disorder was the most common class among the patients. The median duration of the symptoms of allergic rhinitis in the patients was 24 months, and all the patients were diagnosed with moderate to severe allergic rhinitis. Patients with concomitant AR and tic disorder had more frequent AR symptoms and common cold episodes compared to the control group. Allergic and immunological conditions, such as asthma in motor tics, history of food allergy in infancy, history of infantile eczema, and history of previous infections, as well as infantile infections, were also common among patients with vocal tics. These findings emphasize that chronic and severe AR symptoms could lead to tic disorders and the possible association of tic disorders with immunological pathways.

## Data Availability

The datasets used and/or analyzed during the current study are available from the corresponding authors upon reasonable request.

## Abbreviations

AR: allergic Rhinitis
DSM-5: 5th edition of Diagnostic and Statistical Manual of Mental Disorders
ARIA: Allergic Rhinitis and its Impact on Asthma
